# Binding of β-lactoglobulin to three phenolics improves the stability of phenolics studied by multispectral analysis and molecular modeling

**DOI:** 10.1016/j.fochx.2022.100369

**Published:** 2022-06-15

**Authors:** Shanying Zhang, Xiaolei Li, Binling Ai, Lili Zheng, Xiaoyan Zheng, Yang Yang, Dao Xiao, Zhanwu Sheng

**Affiliations:** a Haikou Experimental Station, Chinese Academy of Tropical Agricultural Sciences, Haikou 570101, China; b College of Food Science and Engineering, Hainan University, Haikou 570228, China; c Haikou Key Laboratory of Banana Biology, Haikou 571101, China

**Keywords:** β-Lactoglobulin, Phenolics, Stability, Interaction mechanism, Muti-spectroscopy, Advanced glycation end product

## Abstract

Phenolics have been used to suppress the formation of advanced glycation end products (AGEs) in food; however, enhancing their thermostability and photostability in foods remains a key issue. Ferulic acid (FA), quercetin (QT), and vanillic acid (VA), which reduce production of AGEs, were embedded in bovine β-lactoglobulin (β-LG) and their interaction mechanism was investigated. Fluorescence experiments demonstrated that FA and QT displayed typical static quenching, while VA caused fluorescence sensitization of β-LG. Furthermore, phenolics changed the secondary structure of β-LG by inducing the transformation from α-helices to β-structures, with Van der Waals forces and hydrogen bonds as the primary underlying forces. The thermal and photostability of FA/QT/VA was significantly improved upon binding to β-LG. Furthermore, QT, FA and VA demonstrated good AGEs inhibitory abilities in BSA-fructose, BSA-MGO, arginine-MGO models. These results reveal that β-LG embedding effectively improves the thermostability and photostability of dietary phenolics in food.

## Highlights

• Ferulic acid (FA), quercetin (QT), and vanillic acid (VA) can reduce AGEs production.
• Phenolics changed the secondary structure of β-lactoglobulin (β-LG).
• The binding pattern were explored by molecular docking, molecular dynamic simulation.
• β-LG embedding improves the thermostability and photostability of dietary phenolics.

## 1 Introduction

Advanced glycation end products (AGEs) are a category of harmful products formed by a series of complex reactions during non-enzymatic Maillard reactions (Luevano-Contreras & Chapman-Novakofski, 2010). Numerous researches have revealed that foods produce large amounts of AGEs during processing and storage, of which approximately 10 % enter the human circulatory system after consumption. Although one-third of these AGEs are excreted via the kidneys, those that remain in the body can induce cancer and chronic diseases, like diabetes mellitus and nephropathy (Vlassara & Striker, 2013). Thus, it is important to control dietary AGEs to improve food safety and prevent related diseases.

Dietary phenolics have been proven to be effective an effective inhibitor of the formation of AGEs in foods (Khan et al., 2020, Pu et al., 2021). Previously, we demonstrated that phenolics can decrease AGEs formation in food by scavenging free radicals, inhibiting the formation of key enzymes (α-glucosidase and α-amylase) active for AGEs, and reacting with active dicarbonyl compounds (acetal aldehyde) to form additive products (Shen et al., 2017, Sheng et al., 2018; Sheng et al., 2019). However, prolonging storage time of phenolics can weaken these inhibitory effects, deepen the color and reduce the hardness of food (Sheng, Gu, Hao, Shen, & Zhang, 2016), and even alter the flavor of food (Wang et al., 2017). Furthermore, the antioxidant capacity of fortified dietary phenolics decreases during food processing due to thermal degradation and transformation (Zhang, Feng, & Wang, 2014). Therefore, it is pretty important to improve the thermal and light stability of phenolics in foods.

Quercetin (QT), Ferulic acid (FA) and Vanillic acid (VA) are the most abundant phenolics present in many fruits, flowers, vegetables, and herbs, frequently used as flavoring and scenting agent in the food industry. QT, FA and VA have been reported to exert anti-inflammatory, antioxidative, antidiabetic, anti-Alzheimer’s disease, and antidepressant activities (Chuang, Wei, Lin, & Li, 2020). Furthermore, QT, FA and VA can inhibit AGEs formation, the antiglycation mechanisms of QT, FA and VA involve directly trapping methylglyoxal and glyoxal, inhibiting DNA glycation and α-dicarbonyl compound-induced protein glycation, scavenging reactive oxygen species (ROS), altering the conformation and microenvironment of proteins, and inhibiting α-amylase/α-glucosidase activity via mixed/non-competitive, etc. (Li et al., 2014, Zhang et al., 2014, Zheng et al., 2020). For example, QT inhibits AGEs formation by reducing plasma and tissue methylglyoxal concentrations in healthy mice (Zhao, Tang, & Sang, 2021). Moreover, VA in baijiu vinasse extract suppressed the production of N^ε^-carboxymethyllysine by trapping and eliminating glyoxal, which in turn inhibited the production of AGEs (Wang, Liu, Zhang, Wang, Wang, & Sun, 2019).

An increasing number of studies have indicated that the complexation of phenolics with proteins can contribute to their sustainability and bioavailability (Liu et al., 2021, Liu et al., 2021, Lu et al., 2016, Simes et al., 2020, Zhan et al., 2020). β-Lactoglobulin (β-LG) is one of the major whey protein that contains several binding sites and thus can bind to various ligands with different types (Paul, Ghosh, & Mukherjee, 2014). The specific or nonspecific combination of proteins to phenolic compounds improves the stability of the phenolic (Lu et al., 2016). For instance, β-LG can simultaneously bind to folic acid, resveratrol, and α-tocopherol to form a protein multiligand complex that reduces the oxidative degradation of α-tocopherol and the photodegradation of folic acid (Zhang, Liu et al., 2014). Although the antiglycation activity of phenolics has been remarkably described in chemical model and in *vitro*, few research have investigated the effect of β-LG binding to a single ligand on thermal and light stability or β-LG structure.

In the present study, β-LG was employed as a carrier protein for the ligands FA, QT, and VA, all of which are known to inhibit AGEs formation. The binding mechanism between β-LG and FA/QT/VA was examined using fluorescence spectroscopy, isothermal titration calorimetry (ITC), circular dichroism (CD), and Fourier transform infrared spectroscopy (FTIR), while docking studies were used to analyze changes in the secondary structure of β-LG after binding and high-performance liquid chromatography (HPLC) was used to analyze changes in the stability of FA/QT/VA combined with β-LG. The findings of this study improve our understanding of the binding interaction between β-LG and phenolics, and may useful for the development of an effective inhibitor of AGEs for the food industry.

## 2 Materials and methods

### 2.1 Materials and chemicals

Bovine β-LG (purity ≥ 90 %, molecular weight 18 276 Da), VA (purity ≥ 97 %), QT (purity ≥ 96 %), FA (purity ≥ 99 %), and aminoguanidine hydrochloride (AH; purity ≥ 98 %) were purchased from Sigma-Aldrich (St. Louis, MA, USA). β-LG and FA solutions were prepared with 0.05 mol/L phosphate buffered saline (PBS; pH 7.4). QT and VA solutions were prepared with 75 % ethanol. All other chemicals were of analytical grade.

### 2.2 Ligand antiglycation capability

The antiglycation capability of FA, QT, and VA (the same concentration of AH solution as the positive control group) was determined using BSA-fructose, BSA-MGO, and arginine-MGO models, as mentioned by Shen et al. (2017).

### 2.3 Fluorescence spectroscopy analysis

All fluorescence spectra were acquired according to Zhang et al. (2022). A β-LG stock solution (10 μmol) was prepared in PBS with NaN_3_ (0.02 % w/v) as a preservative. An FA solution was freshly prepared in PBS, while QT and VA were dissolved in ethanol and diluted with PBS to the designated concentration and a final ethanol concentration of 2.5 % (v/v). First, 0.4 mL of β-LG solution (10 μmol) was added into a cell with different volumes of FA/QT/VA solutions (200 μmol) and PBS (0.05 mol/L, pH 7.4) to obtain 4 mL samples with various molar ratios (FA/QT/VA: β-LG, 0, 2, 4, 6, 8, 10, 12, 14, 16, and 18). The fluorescence excitation-emission matrix (EEM) spectra were set according to the method of Jia et al. (2017).

### 2.4 Fluorescence quenching analysis

The quenching mechanism and thermodynamic parameters between the protein and ligands were studied according to the Stern-Volmer’s equation (Lakowicz, 2006), as follows:

(1) $$\frac{F_{0}}{F}=1+K_{q}\tau_{0}\left[Q\right]=1+K_{\mathrm{SV}}\left[Q\right]$$

where *F* are the fluorescence intensities with a quencher, *F_0_* are the fluorescence intensities without a quencher, *K_q_* is the biomolecular quenching rate constant, *τ_0_* is the average lifetime of the biomolecule without a quencher (τ_0_ = 10^-8^ s), [*Q*] is the quencher concentration, and *K_SV_* is the Stern-Volmer quenching constant.

For static quenching, the binding constant (*Ka*) was used for the interaction between the quenchers and protein, with the number of binding sites per protein (*n*) calculated using the following double-logarithm equation:

(2) $$\text{log}\frac{F_{0}-F}{F}=\text{lo}gK_{a}+n\log\left[Q\right]$$

The van’t Hoff equations were used to calculate the entropy change (*△S*), enthalpy change (*△H*), and free energy change (*△G*), as follows:

(3) $$\ln K_{a}=\frac{-\Delta H}{\mathrm{RT}}+\frac{\Delta S}{R}$$

(4) ΔG=ΔH-TΔS

where *T* is the experimental temperature and *R* is the gas constant (8.314 J mol^−1^ K^−1^).

### 2.5 UV–vis absorption spectroscopy analysis

The UV–vis absorption spectra of 3 mL β-LG (10 μmol) and β-LG-FA/QT/VA complexes (10 μmol, c(FA/QT/VA:c(β-LG) = 1:1) were recorded using an Evolation 300 spectrometer (Thermo Fisher Scientific, MA, USA) in quartz cuvette. PBS (0.05 M, pH 7.4) was used as a blank. UV wavelengths ranged from 200 to 450 nm (2 nm slit width) (Jia, Gao, Hao, & Tang, 2017).

### 2.6 ITc

ITC measurements were taken using a Nano ITC (TA, Thermo Fisher Scientific). β-LG and FA/QT/VA solutions were prepared as before and vacuum degassed 10 min before use. Next, 300 μL of β-LG solution (30 μmol) was loaded into the calorimeter sample cell, and 50 μL of FA/QT/VA solution (600 μmol) was loaded into the injection syringe. Protein was titrated with FA/QT/VA in a sequence of 20 aliquots (2 μL), at 200 s intervals to equilibrate. The temperature of the mixture in the titration cell was set at 298 K and the cuvette was continuously agitated at 300 rpm/min during the whole experiment. Integrate the original data as a graph of heat (mcal/s) and time (s) to obtain the observed enthalpy change curve (*△H,* kcal mol^−1^) and molar ratio per mole of injection. PBS was titrated with 600 μmol FA/QT/VA as a control. The experimental data were corrected by subtracting the FA/QT/VA values from the buffer control values and analyzed using the Nano ITC data analysis program (Thermo Fisher Scientific). Gibbs free energy was calculated using Equation 4 (Zhan et al., 2020).

### 2.7 FTIR spectroscopy analysis

The FTIR spectra of the β-LG solution (0.1 mmol) were obtained using a Nicolet 6700 spectrometer (Thermo Fisher Scientific) at wavenumber of 1000 to 4000 cm^−1^ with a 4 cm^−1^ resolution and 32 scans. FA/QT/VA (0.1 mmol) and PBS were loaded to the 0.1 mmol/L β-LG solution to acquire a β-LG: FA/QT/VA molar ratio of 1:1. Unreacted phenolics were dialyzed (molecular weight cutoff, 10 KDa) for 12 h at 4℃, with ultra-pure water changed ten times every 3 h. PBS was used as a blank. The β-LG and β-LG-FA/QT/VA complex secondary structures were analyzed in the 1600–1700 cm^−1^ spectral region containing the amide-I absorption band of the peptide backbone (Zhang, Liu et al., 2014). The numbers and locations of the component bands, the relative percentages of secondary structure elements were calculated using PeakFit software version 4.12 (Stanic-Vucinic et al., 2012).

### 2.8 CD spectroscopy analysis

CD spectra were obtained by a Chirascan CD spectrometer (Applied PhotoPhysics, Leatherhead, UK). The wavelength ranged from 180 to 260 nm with a response time of 4 s three times per spectrum. The β-LG and β-LG- FA/QT/VA complexes (20 μmol) were at a molar ratio of 1:1. The secondary structural components of all compounds were analyzed using the SELCON3 algorithm of CDPro.

### 2.9 Molecular docking

The molecular docking of each ligand (FA, QT, and VA) with β-LG (PDB ID: 2Q2M; https://www.rcsb.org/pdb) was analyzed with AutoDock version 4.2.6 (Morris et al., 2009). The initial structure preserving the original protein charge was prepared using AutoDock Tools version 1.5.6 and a pdbqt file was generated for docking analysis. The 3D structures of the ligands were downloaded from the PubChem database (Figure S1). Since the experiments were performed under neutral conditions, the carboxyl groups in the FA and VA structures were deprotonated. The whole protein was wrapped in a docking box with central coordinates (38.75, 54.25, 25.59). The number of XYZ grid points in the box was 126 × 126 × 126 with grid spacing of 0.375 Å and 200 GA runs, with the other parameters were set to default. The structure with the lowest docking energy was subjected to energy minimization using an Amber14SB forcefield (Maier et al., 2015). Two rounds of optimization were carried out: 1) 2000 steepest descent steps; 2) 2000 conjugate gradient steps.

### 2.10 Molecular dynamics simulation

The molecular dynamics simulation of β-LG with or without ligands systems were carried out with YASARA (Krieger, Dunbrack, Hooft, & Krieger, 2012). YASAR was able to optimize the environment for solute stability and the protonated state of protein residues. After steepest descent and simulated annealing was minimized to eliminate conflicts, the simulation was run for 100 ns with the AMBER14 force field (Maier et al., 2015) for the solute, GAFF2 and AM1BCC for ligands and TIP3P for water. Other parameters such as Van der Waals forces, Long-range electrostatics, temperature and pressure were set according to the method of Krieger & Vriend (2015).

### 2.11 Photostability of FA/QT/VA solutions under visible light

FA/QT/VA samples with and without β-LG were exposure by irradiation under visible light using LS-4000 illumination machine (Tianxing Keyi Corporation, Beijing, China). Illuminance was adjusted to 4000 Lx and the temperature was maintained at 25 °C. Samples were placed approximately 10 cm away from the light source and analyzed every 6 h for up to 36 h. Control samples were placed in the same environment but protected from light. To separate the free ligands, 0.4 mL of β-LG-FA/QT/VA,

The solution was dialyzed with a molecular weight of 3 kDa in a dialysis bag (Merck Millipore, Ireland) and centrifuged at 12,000×*g* for 15 min at 4 °C. Changes in the FA/QT/VA content of the solutions were determined by injecting exactly 10 μL of each solution into an HPLC system (ACQUITY UPLC I-Class, Waters, USA) fitted with a Waters BEH C18 column (2.1 × 50 mm, 1.7 μm). The relevant assay was referred to the method of Zhang et al. (2022).

### 2.12 Thermal stability of FA/QT/VA solutions

FA/QT/VA samples with and without β-LG were placed in water baths at 25 (control), 35, 45, and 55 °C in the dark. After 8 h, the samples were removed from the water baths and the phenolic content of the samples was assayed by HPLC, as described in section 2.11.

### 2.13 Statistical analysis

All experiments were repeated in triplicates. Data analysis and mapping were processed by the softwares of Origin 8.5 software. Statistical analyses were performed using SPSS 19.0 software (SPSS). Analysis of variance (AVONA) and Tukey's significant difference test after least significant, *P* ≤ 0.05.

## 3 Results and discussion

### 3.1 Antiglycation capability of FA/QT/VA

**Fig. 1:**
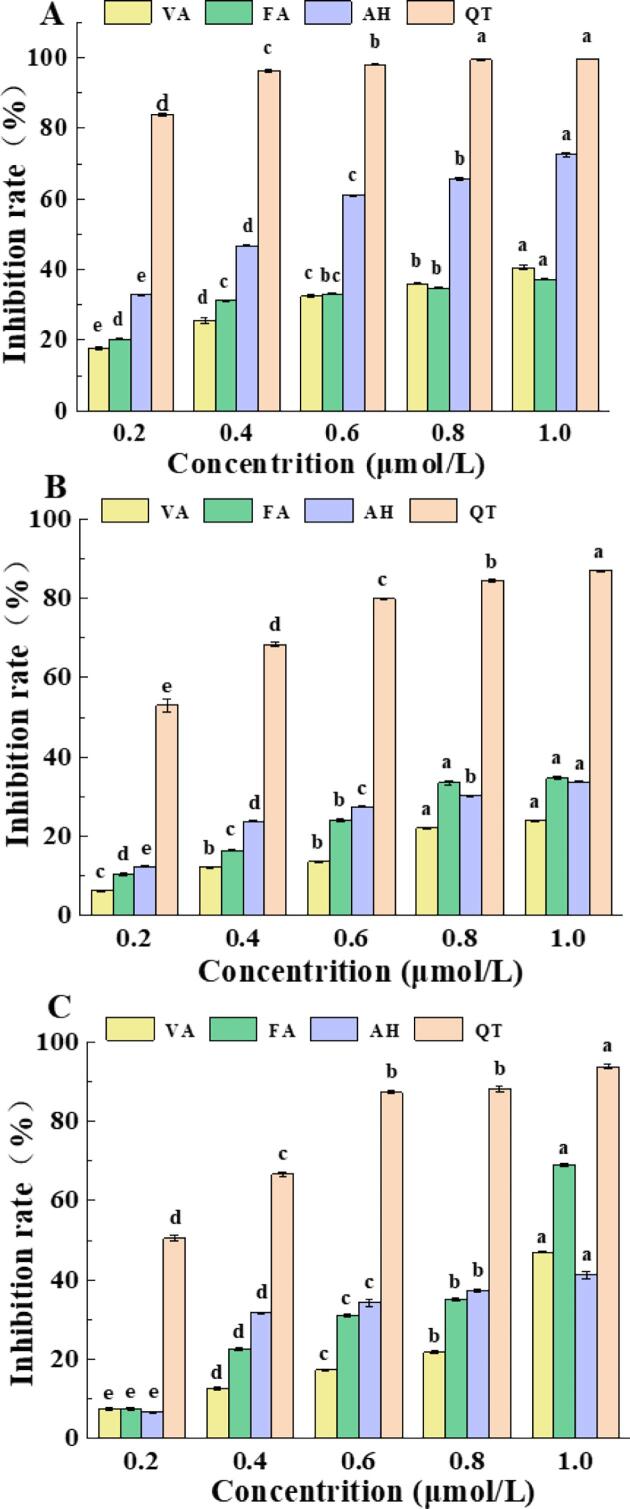
Inhibition rate of ferulic acid/quercetin/vanillic acid on AGEs in BSA-fructose (A), BSA-acetone aldehyde (B) and arginine-acetone aldehyde (C) models (Different lowercase letters under the same inhibitor represent p < 0.05, otherwise p > 0.05).

The BSA-fructose model was used to assess the inhibition of AGEs formation and reflects the BSA antiglycation capability of the ligand (Shen et al., 2017). As shown in Fig. 1A, all ligands dose-dependently inhibited AGEs formation, in the order of QT > AH > FA > VA. The better ability of these three ligands to inhibit AGEs formation may be partly attributed to their excellent ROS and ligand-MGO conjugate scavenging ability (Li et al., 2014, Khan et al., 2020, Wang et al., 2019). At all testing concentrations, QT displayed a higher inhibition rate of AGEs than AH, artificial compound with the potential to treat diabetic comorbidity, suggesting that QT is a compound with great potential for scavenging AGEs. This is due to the ability of QT to inhibit glycation-induced changes in conformational structure and microenvironment, which in turn inhibits BSA glycation. Moreover, QT can inhibit cross-linking or aggregation of glycated BSA, altering the glycation site of BSA (Zhang et al., 2019, Zhang et al., 2019).

The BSA-MGO model was developed to simulate the intermediate stage of protein glycation, where the ligand-containing reaction solution dose-dependently increased AGEs inhibition capability. When the ligand concentration increased from 0.2 to 1 mmol (Fig. 1B), AGEs inhibition increased significantly from 10.38, 53.05, and 6.32 % to 34.75, 87.02, and 23.05 %, respectively. These findings could be explained by the fact that QT is more selective than FA and VA in targeting MGO and can prevent MGO inducing protein crosslinking (Lin, Tan, Lai, & Zhou, 2018). Indeed, Li et al. (2014) showed that QT can efficiently trap MGO and reduce AGEs formation by forming mono- and di-MGO adducts.

Arginine can readily attack MGO during glycation to promote the production of irreversibly modified proteins. In particular, MGO reacts with the guanidine group of arginine to product argpyrimidine. Thus, the antiglycation ability of ligands could be assessed using an arginine-MGO model (Shen et al., 2017). When the QT concentration exceeded 0.6 mmol, its antiglycation inhibition rate was over 80 % (Fig. 1C). The anti-glycation ability of QT or VA was markedly higher than that of AH or FA at all test concentrations except for 1.0 mmol.

Overall, QT displayed the highest AGE inhibition rate in the three models, with the strongest AGE inhibition (up to 99.56 %) in the BSA-fructose model. At higher concentrations, FA and VA also demonstrated good AGEs inhibitory abilities in all three models.

### 3.2 Interaction between β-LG and FA/QT/VA

**Fig. 2:**
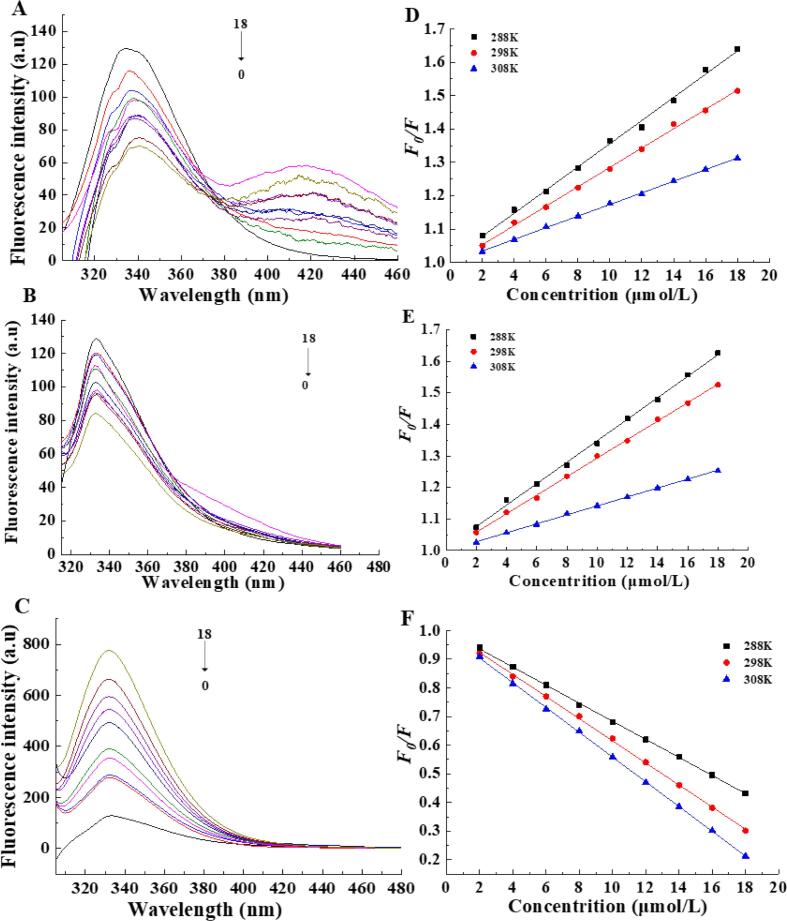
Intrinsic fluorescence of β-LG-FA(A)/QT(B)/VA(C) complexes (c(β-LG B) = 1 μmol, c(FA/ QT/ VA): c(β-LG) = 0, 2, 4, 6, 8, 10, 12, 14, 16, 18, T = 298 K, pH = 7.4, λex = 295 nm).Stern-Volmer plots of the interaction between β-LG and FA (E)/QT(F)/VA(C) at different temperatures (pH = 7.4, λex = 295 nm).

Fluorescence spectroscopy is a validated method for evaluating the ligand–protein interactions (Jia et al., 2017, Zhang et al., 2014) since changes in the protein microenvironment and conformational transition by ligand binding can alter the intrinsic fluorescence of a protein (Lakowicz, 2006). The binding of phenolics to proteins will change the fluorescence excitation state of proteins (Wang, Xu, Han, & Tang, 2021). The intrinsic fluorescence of β-LG is derived from two tyrosine and four tryptophan residues, with tyrosine 19 being the main (80 %) contributor in an apolar environment (Liang and Subirade, 2010, Liang et al., 2011). In this study, the β-LG spectrum exhibited the maximum emission wavelength at 334 nm (Fig. 2A-C), which was consistent with the result of a previous study (Liang et al., 2011). Its intrinsic fluorescence intensity decreased with increasing FA/QT concentration (Fig. 2A, B). It suggested that FA and QT interacted with β-LG amino groups to exert their shielding effects (Wang et al., 2019). The fluorescence quenching ability of QT against β-LG was significantly greater than that of FA, with a slight red shift (Fig. 2A). It indicated that one or both Try residues moved to a more polar micro-environment via secondary structural alterations upon FA addition (Abdollahi, Condict, Hung, & Kasapis, 2020). Conversely, the intrinsic fluorescence of β-LG increased with increasing VA concentration without altering the maximum emission wavelength (Fig. 2C). Comparing the fluorescence spectra of β-LG-FA and β-LG-VA with the chemical structures of FA and VA revealed that both had the same amount of hydroxyl groups. Thus, the numbers and positions of phenolic hydroxyl groups may affect their β-LG fluorescence quenching ability, while their structure may influence the type of β-LG fluorescence change (Li et al., 2020).

### 3.3 Fluorescence quenching and sensitization mechanisms and binding constant

Reduced fluorescence intensity can be attributed to various phenomena, including excited-state reactions, energy transfer, molecular rearrangements, ground state complex formation, and collisional quenching (Lakowicz, 2006, Liu et al., 2021). Changes in fluorescence between substances and solvents can be classed as either static or dynamic. For instance, static fluorescence quenching often changes the absorption spectrum of the static complex and reduces the quenching constant as a result of decreasing complex stability with increasing temperature. During dynamic quenching, excited fluorescence quencher molecules collide with the quencher and affect the excitation state of the fluorescent substance but do not change its absorption spectrum (Bi, Pang, Wang, Zhao, & Yu, 2014; Mulvihill & Kinsella, 2010). These principles also apply to fluorescence sensitization (Zhan et al., 2020).

In this study, β-LG fluorescence quenching and sensitization using the equation 1 at three different temperatures (288, 298, 308 K) were analyzed. VA caused β-LG fluorescence sensitization with a negative fluorescence quenching constant and fluorescence quenching rate constant; however, as the temperature increased, β-LG quenching (*K_SV_*) decreased (Fig. 2D, E and Table S1). The ligand quenching rate constant calculated (Equation (2)) from the measured *Ksv* values revealed that the *K_q_* was higher than the maximum diffusion collision quenching constant (2.0 × 10^10^ L/mol/s) and indicating that FA and QT display typical static quenching whereas VA displays sensitization (Zhan et al., 2020). The amount of binding sites (*n*) approached 1 when FA/QT/VA existed, which indicated a solitary binding site on β-LG. The *K_a_* of FA was greater than that of QT at 298 K and decreased with increasing temperature for both the β-LG-FA and β-LG-QT complexes. It suggested that their binding reaction between FA/QT and β-LG was exothermic. Conversely, the *K_a_* of VA rose with increasing temperature (Fig. 2F) suggested that the binding process between VA and β-LG was endothermic, and the above results were consistent with the finding of Xu et al. (2019). These findings may demonstrate that VA binds with β-LG in a different manner to FA and QT. Furthermore, the UV–vis absorption spectrum of β-LG with a significant red shift after binding to FA/QT/VA (Fig. 1) indicated that they altered the microenvironment of the β-LG chromophore (Lakowicz, 2006) and confirmed that FA/QT/VA binding caused β-LG static quenching or sensitization.

### 3.4 Thermodynamic characterization o

**Fig. 3:**
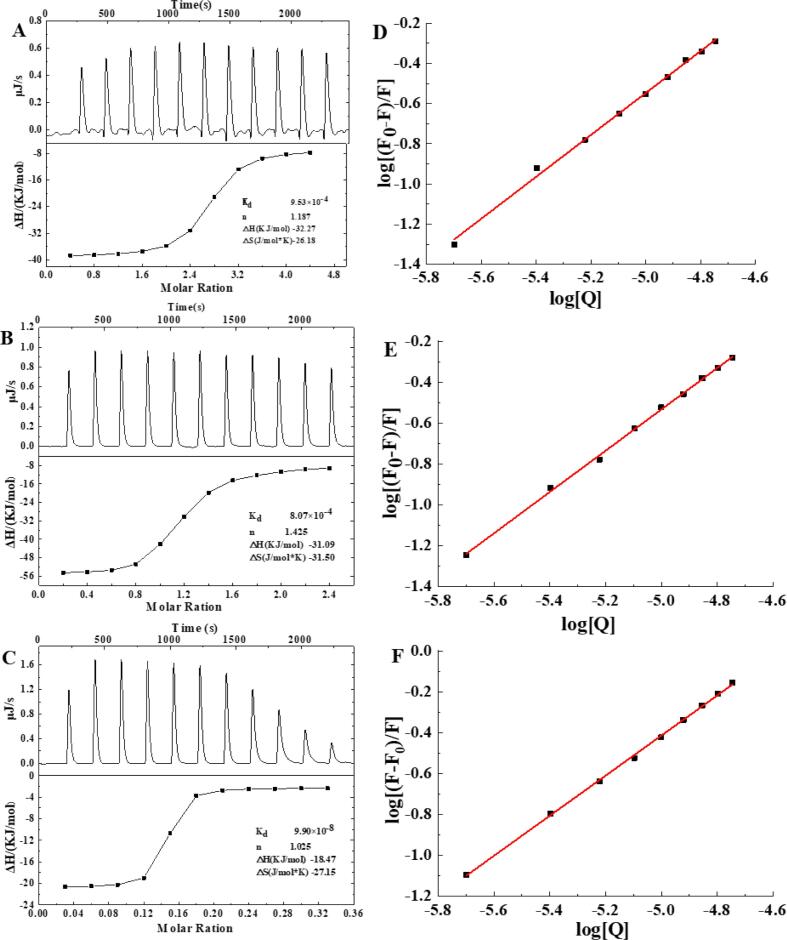
ITC of FA(A)/QT(B)/VA(C) (600 μmol) titration of β-LG (30 μmol); molar enthalpy change (, kcal/mol) against the molar ratio of the total FA(A)/QT(B)/VA(C) to β-LG after subtracting the control experiment. Double logarithmic plot of changes in fluorescence intensity as a function of FA(D)/QT(E)/VA(F) concentration (0–18 μmol, pH = 7.4, T = 298 K).

ITC can sensitively probe enthalpy changes associated with the interactions between β-LG and FA/QT/VA (Zhan et al., 2020). The *△S*, *△H*, and *△G* were determined in a single ITC experiment (Fig. 3A-C) and calculated using Equations (3) and (4) (Fig. 3D-F). There are four forms of protein–ligand interactions: electrostatic interactions, hydrophobic interactions, van der Waals forces, and hydrogen bonds (Bi et al., 2014). When *△H* > 0 and *△S* > 0, hydrophobic effects are the main force; when *△H* < 0 and *△S* < 0, van der Waals forces and hydrogen bonds are the main forces; and when *△H* < 0 and *△S* > 0, electrostatic forces are the main interaction (Ross & Subramanian, 1981). The data shown in Table S2 and Fig. 3, the calculated *△S* value was negative and the amount of binding site of one, suggested that van der Waals forces and hydrogen bonds were the primary forces between FA/QT/VA and β-LG, while the negative △*G* values suggested that the binding process between FA/QT/LG and β-LG was spontaneous (Xu et al., 2019). Although the calculated and determined values were different, the qualitative analysis revealed that there was consistently only one FA/QT/VA binding site on β-LG, with van der Waals forces and hydrogen bonds being the primary forces involved.

### 3.5 Fluorescence EEM spectra

**Fig. 4:**
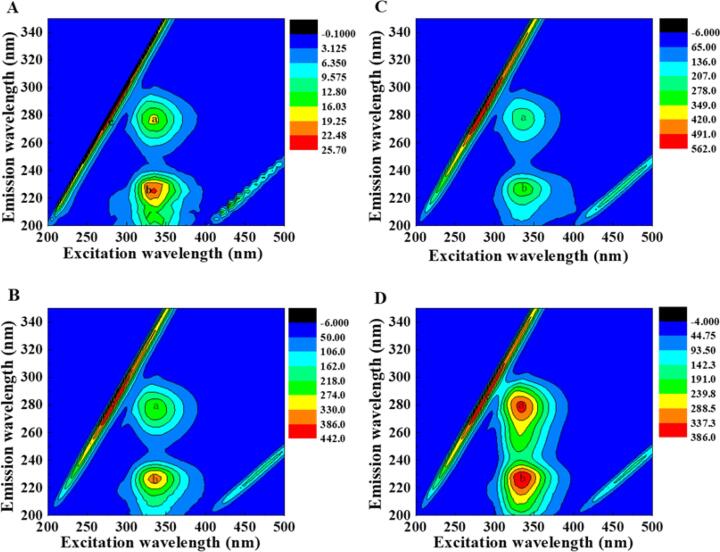
Fluorescence EEM landscapes of β-LG (A) and the β-LG-FA (B)/ QT (C) /VA (D) complexes. C (β-LG) = 1.0 μmol, c (FA)/c(QT)/c (VA): c (β-LG) = 2:1.

Fluorescence EEM spectra are an effectively way to detect protein conformational changes in detail. The results of fluorescence EEM spectra of β-LG in the presence and absence FA/QT/VA are shown in Fig. 4A-D, in which peak *a* referred to the spectral characteristics of tyrosine and tryptophan residues, peak *b* the polypeptide backbone structure (intensity related to protein secondary structure) (Bi et al., 2014). After adding FA/QT, peaks *a* and *b* decreased (Fig. 4A-C) and indicated a strong interaction between β-LG and FA/QT. Conversely, VA increased the fluorescence intensity of peaks *a* and *b* (Fig. 4D). It suggested that VA may alter β-LG protein secondary structure and conformation Jia et al. (2017) analyzed conformational changes in β-LG bound to phenolics using 3D fluorescence spectroscopy and found that the extent of peak *a* and *b* quenching correlated with a decreasing number of phenolic hydroxyl groups (epigallocatechin-3-gallate > FA > chlorogenic acid). Consequently, the degree of peak *a* and *b* quenching was assumed to be associated with the number of phenolic hydroxyl groups. QT has more hydroxyl groups than FA. Thus, it had greater degree of fluorescence quenching; while FA and VA both have the same number of hydroxyl groups. The changes in peaks *a* and *b* are also related to phenolic structure (Li et al., 2020).

### 3.6 Effect of phenolic binding on β-LG secondary structure

FTIR is commonly used to evaluate changes in protein secondary structure, particularly the amide I band between 1600 and 1700 cm^−1^. In this study, we estimated the secondary structure of β-LG by fitting the peaks of the amide I band. Generally, the peaks between 1600 and 1639 cm^−1^ represents β-fold, 1640–1650 cm ^-1^ represents irregular curls, 1651–1660 cm^−1^ represents α-helices, and 1661–1700 cm^−1^ represents β-turns (Stanic-Vucinic et al., 2012, Ren et al., 2022). The fit peak of β-LG changed significantly after binding to FA/QT (Figure S2A-D), for example the 1600–1639 cm ^-1^ peaks increased significantly and the 1651–1660 cm ^-1^ peaks decreased. In particular (Table S2), α-helices were reduced by 4.61 % and 4.46 % after binding to FA/QT, while β-sheets increased by 3.80 % and 3.59 %, respectively. The peaks between 1640 and 1650 and 1661–1700 cm ^−1^ significantly decreased whereas those between 1600 and 1639 cm ^−1^ significantly increased (Figure S2B, D). Thus, the α-helix in β-LG did not change significantly after binding to VA, whereas the β-sheet increased by 4.87 % and the β-turn and random coil decreased by 2.78 and 2.02 %, respectively (Table S2).

Since peak fitting is associated with uncontrollable errors, structural changes in β-LG were also studied using a CD spectrometer (Paul et al., 2014) and analyzed using CDPro software (Table S2). After binding to FA/QT, α-helices decreased and β-sheets increased, whereas β-turns and random coils decreased and β-sheets increased after binding to VA. Although the CD and FTIR results were different numerically, both of them were agreement with that β-LG-FA/QT/VA complex formation changed the secondary structure of β-LG.

### 3.7 Molecular docking analysis of binding between β-LG and FA/QT/VA

**Fig. 5:**
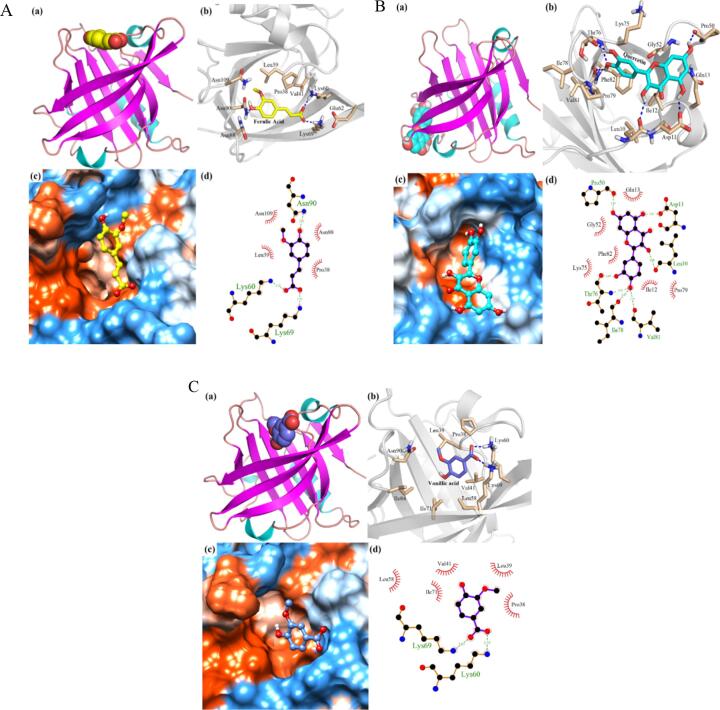
The binding model diagram of FA (A), QT (B), VA(C) and β-LG (Three-dimensional binding mode of FA/QT/VA to β-LG (a); the interaction among VA and surrounding amino acid residues, the blue dotted line indicates the hydrogen bond interaction (b); the binding of VA to the hydrophobic surface of the β-LG, the blue and orange parts represent the hydrophilic and hydrophobic parts of the β-LG, respectively (c); the two-dimensional binding mode between VA and β-LG, the green dotted line indicates the hydrogen bond interaction, and the red gear shape indicates the hydrophobic interaction (d)). (For interpretation of the references to color in this figure legend, the reader is referred to the web version of this article.)

In present study, the precise binding sites between β-LG and FA/QT/VA were simulated by molecular docking analysis to visualize and further understand the interactions of β-LG and FA/QT/VA. As shown in Fig. 5A-C, the FA binding site was located in the hydrophobic pocket at the top of the β-barrel of β-LG. QT bound to the bottom pocket of the β-LG's β-barrel structure, whereas VA bound to the top of the β-bucket of β-LG.

A variety of non-covalent interactions are engaged in the binding of phenolics to proteins, including hydrogen bonding, van der Waals interaction, π-π stacking interaction and hydrophobic interaction. Binding is a result of the interaction between FA/QT/VA and amino acid residues. According to the docking results (Fig. 5A-C), the binding interactions between β-LG and FA/QT/VA involved nine (Pro38, Leu39, Val41, Lys60, Glu62, Lys69, Asn88, Asn90, and Asn109), twelve (Leu10, Asp11, Ile12, Gln13, Pro50, Gly52, Lys75, Thr76, Ile78, Pro79, Val81, and Phe82), and nine (Pro38, Leu39, Val41, Leu58, Lys60, Lys69, Ile71, Ile84, and Asn90) amino acid residues, forming three (Asn90, 2.93 Å; Lys60, 2.72 Å; Lys69, 2.73 Å), seven (Pro50, 2.80 Å; Asp11, 2.82 Å; Leu10, 2.99 Å; Thr76, 2.69/3.01 Å; Ile78, 2.87 Å; Val81, 3.30 Å), and two (Lys69, 2.67 Å; Lys60, 2.75 Å) hydrogen bonds, respectively. Compared with FA and VA, the QT structure has plenty of hydroxyl groups, which form more hydrogen bonds with the surrounding amino acid residues. Three, six and five amino acid residues of β-LG are engaged in the hydrophobic interaction with FA/QT/VA. Van der Waals force is another important non-covalent interaction to maintain the stability of phenolics and β-LG complexes (Meng & Li, 2021). Notably, glutamine and glutamate play an important role in the van der Waals force between flavonoids and β-LG (Pu et al., 2021), such as Glu62 and Gln13. Furthermore, QT formed π-π stacking interaction with Phe82, which enhanced the affinity of QT with β-LG. Therefore, the docking analysis results indicated that van der Waals forces, hydrophobic interaction and hydrogen bonding play significant roles in the interactions among phenolics and β-LG.

### 3.8 Molecular dynamics simulation

Molecular dynamics simulation can be used to calculate the distance of center of mass (DCOM) and binding free energy between FA, QT and VA and β-LG. DCOM can be used to express the distance from the center of mass of phenolics to that of protein (Zhu et al., 2021). As shown in Figure S3A, the DCOM between FA and β-LG decreases from 1.4 nm to nearly 1.0 nm. It indicated that FA gradually extended into the hydrophobic pocket of β-L, forming a more stable FA-β-LG compound. However, the stable DCOM between QT and β-LG in the process of molecular dynamics simulation, was mainly be attributed to the molecular structure of QT forming hydrogen bonds with surrounding amino acids (Figure S3A), which has a relatively stable binding conformation. However, due to the small molecular structure of VA and relatively weak non-covalent interactions with surrounding amino acid residues, the DCOM between VA and β-LG was not stable during the molecular dynamics simulation.

As shown in Figure S3B, the binding energies of FA, VA and QT to β-LG were-79.79 ± 6.45 kJ/mol, −41.65 ± 8.28 kJ/mol and-27.77 ± 6.84 kJ/mol, respectively. The binding energy of FA to β-LG was the highest, while that of VA to β-LG was the lowest. The higher the negative value of the binding free energy, the easier the ligand binds to the receptor (Ossman, Fabre, & Trouillas, 2016). The weaker interaction energy between QT and VA agrees well with the distance fluctuation in Figure S3A of FA. Therefore, the affinity of the three phenolics to β-LG was in the order of FA > QT > VA.

### 3.9 Effect of complex formation on β-LG and FA/QT/VA stability

**Fig. 6:**
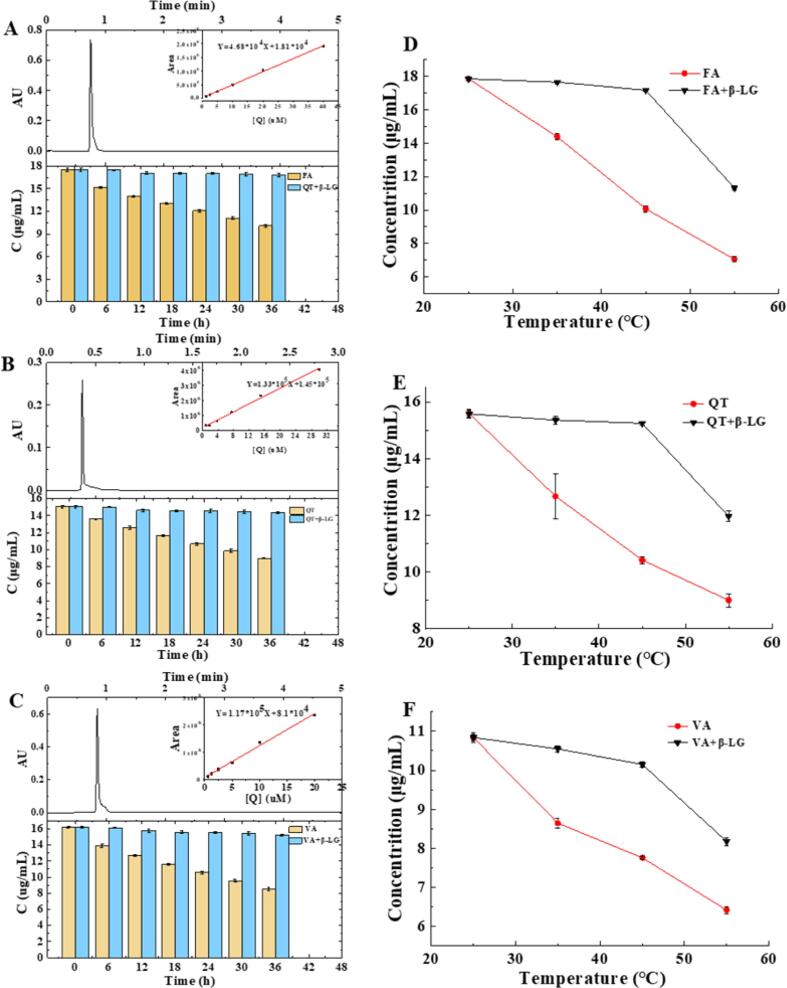
Liquid chromatograms of FA (A)/QT (B)/VA(C) and their changes in the content of the sample under visible light. The content of FA (D)/QT (E)/VA(F) in the sample after 8 h at 25 °C, 35 °C, 45 °C and 55 °C (The initial concentrations of FA/QT/VA in the two experiments were 17.5 μg/mL, 15.1 μg/mL and 15.0 μg/mL, respectively.).

FA is sensitive to ultraviolet radiation which causes it to degrade into *cis*-FA; however, photodegradation of QT and VA have not yet been reported. In this study FA/QT/VA degraded rapidly in the samples without β-LG and their content decreased to 58.4, 59.6, and 52.5 %, respectively, after exposed to visible light irradiation (4000 Lx) for 36 h (Fig. 6A-D). The FA/QT/VA content of the samples containing β-LG did not change significantly and slightly, decreased by 2.9, 4.6, and 6.2 %, respectively. β-LG may have a significant protective effect on FA/QT/VA to slow their photodegradation. Indeed, β-LG denaturation under visible light irradiation may precede and delay FA/QT/VA photodegradation (Zhang, Liu et al., 2014).

In this study, the FA/QT/VA content of the samples containing β-LG was significantly lower than those without β-LG after heat at 35 and 45 °C for 8 h (Fig. 6D-F). Therefore, β-LG may have a significant protective effect on FA/QT/VA at temperatures lower than 45 °C and improve their stability. However, when the temperature exceeded 45 °C, the protective effect of β-LG on FA/QT/VA dropped sharply, due to weakened binding between FA/QT/VA and β-LG at high temperature (Lu et al., 2016).

## 4 Conclusion

This study investigated the binding mechanisms between FA/QT/VA and β-LG using multiple spectroscopic techniques, ITC, and molecular simulation. QT, FA and VA demonstrated good AGEs inhibitory abilities in BSA-fructose, BSA-MGO, arginine-MGO models. FA and QT both displayed typical static quenching with QT exerting a significantly greater quenching ability than FA, while VA caused β-LG fluorescence sensitization. Van der Waals forces and hydrogen bonds were the main forces underlying the spontaneous binding between FA/QT/VA and β-LG. However, CD and FTIR indicated that the complex formation altered the secondary structure of β-LG. After combining with FA/QT, α-helices decreased and β-sheets increased, while β-turns and random coils decreased; and β-sheets increased after combining with VA. The molecular dynamics simulation results reveled that the affinity of the three phenolics to β-LG was in the order of FA > QT > VA. Furthermore, FA/QT/VA preferred to bind to the hydrophobic pocket of β-barrel., Also, the thermal and light radiation stability of FA/QT/VA was significantly improved after binding to β-LG.

## CRediT authorship contribution statement

**Shanying Zhang:** Writing – original draft, Methodology, Investigation. **Xiaolei Li:** Writing – original draft, Data curation. **Binling Ai:** Methodology. **Lili Zheng:** Methodology. **Xiaoyan Zheng:** Investigation. **Yang Yang:** Investigation. **Dao Xiao:** Data curation. **Zhanwu Sheng:** Funding acquisition, Writing – review & editing, Supervision.

## Declaration of Competing Interest

The authors declare that they have no known competing financial interests or personal relationships that could have appeared to influence the work reported in this paper.
