# Supplementary material for: Binding of β-lactoglobulin to three phenolics improves the stability of phenolics studied by multispectral analysis and molecular modeling
Source: Food Chem X. 2022 Jun 15;15:100369. doi: 10.1016/j.fochx.2022.100369 (PMC9234335; doi:10.1016/j.fochx.2022.100369)
Supplement: Supplementary data 1 [file mmc1.docx]

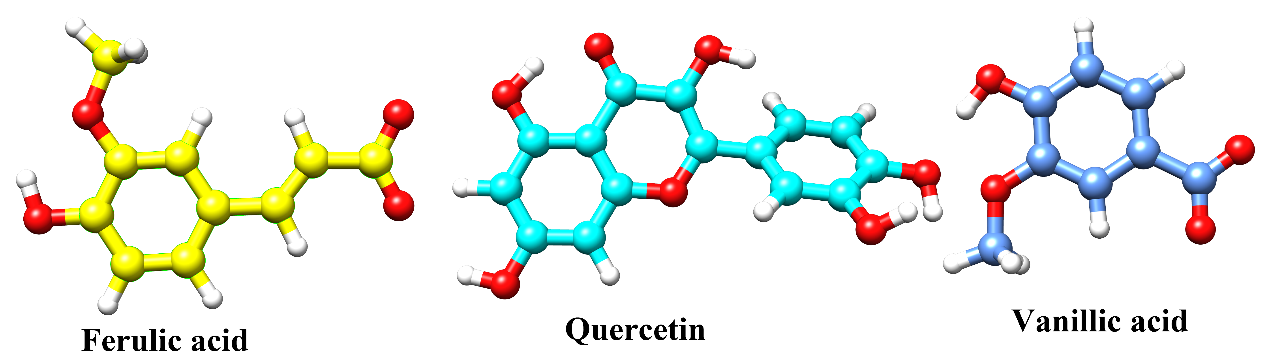


Fig. S1 3D structures of FA, QT and VA.


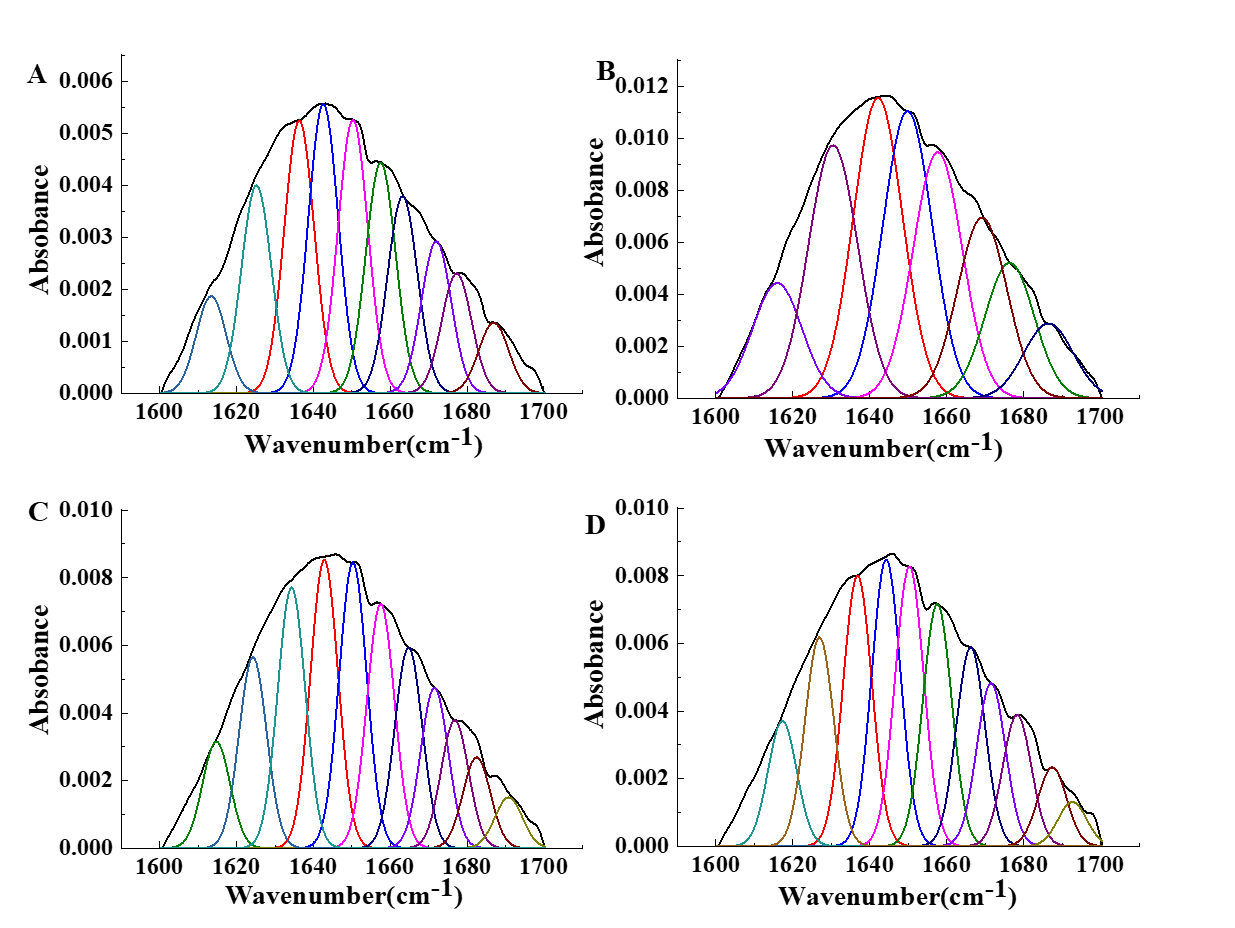


Figure S2 FTIR spectra peak fitting of β-LG B (E) and β-LG B-FA (B)/ QT (C) /VA (D) complexes, c (β-LG B) = 0.1 mM, c (FA)/c (QT)/c (VA): c (β-LG B) = 1:1.

Figure S3 The distance of center of mass (A) and binding energy (B) of FA/QT/VA to β-LG.

Table S1

The quenching and Bind constants and thermodynamic parameters for the interaction of β-LG and FA/QT/VA with MFA (Molecular Fluorescence Analysis) and ITC (Isothermal Titration Calorimetry) at the temperature of 298 K.

| Compound | K_SV_ (10^4^L/mol) | | | n | | K_a_(10^4^L/mol) | | | △H(KJ/mol) | | △G(KJ/mol) | | | △S(KJ/mol/K) | | |
| --- | --- | --- | --- | --- | --- | --- | --- | --- | --- | --- | --- | --- | --- | --- | --- | --- |
|  | MFA | ITC | MFA | | ITC | | MFA | ITC | MFA | ITC | | MFA | ITC | | MFA | ITC |
| β-LG+FA | 3.12 | — | 1.03 | | 1.19 | | 4.65 | 5.24 | -40.1 | -32.27 | | -33.84 | -24.52 | | -0.021 | -0. 026 |
| β-LG+QT | 2.94 | — | 1.01 | | 1.43 | | 3.26 | 2.85 | -27.37 | -31.09 | | -16.94 | -21.55 | | -0.035 | -0. 032 |
| β-LG+VA | -3.82 | — | 0.98 | | 1.03 | | 3.05 | 3.74 | -21.81 | -18.74 | | -16.15 | -20.69 | | -0.019 | -0. 027 |

Table S2

CD and FTIR spectra parameters of β-LG in the absence and presence of FA/QT/VA.

| Compound | α-Helix | | β-Sheet | | β-Turn | | Random. Coli | | |
| --- | --- | --- | --- | --- | --- | --- | --- | --- | --- |
|  | CD | FTIR | CD | FTIR | CD | FTIR | | CD | FTIR |
| β-LG | 15.96% | 16.54% | 38.94% | 45.37% | 21.85% | 17.47% | | 23.25% | 20.62% |
| β-LG+FA | 11.58% | 11.93% | 42.42% | 49.17% | 22.06% | 17.95% | | 23.94% | 20.95% |
| β-LG+QT | 12.23% | 12.08% | 41.57% | 48.96% | 21.94% | 17.29% | | 24.26% | 21.67% |
| β-LG+VA | 15.43% | 16.47% | 44.19% | 50.24% | 18.47% | 14.69% | | 21.91% | 18.60% |
